# Influence of sildenafil on the anticonvulsant action of selected antiepileptic drugs against pentylenetetrazole-induced clonic seizures in mice

**DOI:** 10.1007/s00702-012-0767-1

**Published:** 2012-02-07

**Authors:** Dorota Nieoczym, Katarzyna Socała, Jarogniew J. Łuszczki, Stanisław J. Czuczwar, Piotr Wlaź

**Affiliations:** 1 Department of Animal Physiology, Institute of Biology and Biochemistry, Maria Curie-Skłodowska University, Akademicka 19, PL-20033 Lublin, Poland; 2 Department of Pathophysiology, Medical University of Lublin, Lublin, Poland; 3 Department of Physiopathology, Institute of Agricultural Medicine, Lublin, Poland

**Keywords:** Sildenafil, Epilepsy, Pentylenetetrazole, Clonic seizures, Seizure models, Antiepileptic drugs, Mice

## Abstract

The aim of the present study was to investigate the effect of sildenafil, a selective phosphodiesterase 5 (PDE5) inhibitor, on threshold for clonic seizures in mice. In addition, the effects of sildenafil on the anticonvulsant activity of selected antiepileptic drugs (AEDs), i.e., clonazepam (CZP), valproate (VPA), phenobarbital (PB), ethosuximide (ETS) and tiagabine (TGB), were also evaluated. The subcutaneous pentylenetetrazole (PTZ) test was used to determine the effects of sildenafil on convulsive susceptibility and the anticonvulsant activity of the studied AEDs in mice, while the acute side effects of sildenafil and its combinations with the studied AEDs were evaluated in the chimney test, step-through passive-avoidance task and grip-strength test in mice. Total brain concentrations of AEDs were also determined. Sildenafil (5–40 mg/kg) did not influence the threshold for PTZ-induced clonic seizures in mice, but increased the anticonvulsant activity of ETS in this test without any significant changes in the total brain concentration. The activity of the remaining AEDs was not significantly changed by sildenafil. Neither sildenafil alone nor its combinations with the studied AEDs produced any changes in the motor coordination, long-term memory and muscular strength in mice. Co-administration of sildenafil with ETS in male epileptic patients with co-existing erectile dysfunctions might lead to the pharmacodynamic interactions that may be beneficial for the patients. Combinations of sildenafil with CZP, VPA, PB and TGB appear to be neutral in terms of their influence on seizures.

## Introduction

Sildenafil, a selective phosphodiesterase 5 (PDE5) inhibitor, is the active ingredient of Viagra^®^ used in the treatment of erectile dysfunction of various etiologies. Its pharmacological activity is connected with nitric oxide (NO)/cyclic guanosine monophosphate (cGMP) pathway. Cyclic GMP is considered an important secondary messenger in the brain and its intracellular level is regulated by guanyl cyclase (GC) isoforms, which take part in its synthesis, and by phosphodiesterases (PDEs), which hydrolyze cGMP to guanosine monophosphate (GMP). Inhibition of PDE5 by sildenafil reduces degradation of cGMP, which leads to the relaxation of smooth muscle in the corpus cavernosum and, consequently, increases blood flow into the penis (Uthayathas et al. 2007).

Expression of PDE5 was noted in the central nervous system, especially in the cerebellum, Purkinje cells and striatum (Domek-Łopacińska and Strosznajder 2005). The ability of sildenafil to cross the blood–brain barrier and the presence of PDE5 in different brain areas predispose this drug to exert some direct central nervous system effects, which were previously noted in both humans and rodents. Among the numerous central nervous system effects of sildenafil, antinociceptive potential was noted (Kim et al. 2010; Huang et al. 2010). Moreover, sildenafil modulates memory formation and learning processes (Rutten et al. 2005; Devan et al. 2006). Evidences from animal studies showed that sildenafil stimulated neurogenesis, which might promote recovery after stroke (Zhang et al. 2002; Bednar 2008). Furthermore, dizziness, depression, headache, light-headedness, visual changes, nervousness, insomnia, abnormal dreams and substance abuse behaviors were noted in humans who used sildenafil (Moreira et al. 2000; Crosby and Diclemente 2004).

The effect of sildenafil on convulsant activity was recently extensively investigated. Its proconvulsant activity was first noted in humans (Gilad et al. 2002) and only later in the pentylenetetrazole (PTZ) (Riazi et al. 2006; Nieoczym et al. 2010b; Montaser-Kouhsari et al. 2011) and bicuculline models (Riazi et al. 2006) of clonic seizures in mice. Sildenafil was also reported to reverse anticonvulsant activity of diazepam (Gholipour et al. 2009) and lithium chloride (Bahremand et al. 2010) in PTZ-induced clonic seizure paradigm. Conversely, our previous research showed that sildenafil increased the threshold for electroconvulsions in a dose-dependent manner and intensified anticonvulsant activity of carbamazepine (CBZ), valproate (VPA) and topiramate (TPM) in the maximal electroshock seizure test in mice (Nieoczym et al. 2010a). Another study showed that sildenafil did not change the threshold for afterdischarges, but limited the duration of behavioral seizures and afterdischarges in the amygdala-kindling in rats (Nieoczym et al. 2010b). Moreover, sildenafil did not influence seizures induced by cocaine (Nieoczym et al. 2009).

Sexual dysfunction is one of the most common disturbances in the interictal period in both men and women with epilepsy. In male epileptic patients, decreased libido and potency, loss of sexual desire and erectile dysfunctions have been noted frequently. While 3–9% of healthy men have erectile dysfunctions, 57% of male epilepsy patients experience this (Smaldone et al. 2004). Sexual dysfunctions in people with epilepsy might be caused by many factors, of which the three main ones are noted: epilepsy itself, antiepileptic drugs (AEDs) and psychiatric/psychotic problems (Smaldone et al. 2004; Stimmel and Gutierrez 2006). It was reported that epileptic discharges in the brain might be transmitted through amygdala–hypothalamic pathways, resulting in changes in the release of sexual steroid hormones and in reduced serum concentration of gonadotropins and bioactive testosterone (Herzog 1999; Montouris and Morris 2005). Moreover, endocrinological disorders might be also caused by AEDs, especially by the liver enzyme inducing AEDs, like phenobarbital (PB), phenytoin (PHT) and carbamazepine (CBZ). These drugs enhance synthesis of sex hormone-binding globulins (SHBG) and sexual steroid metabolism in the liver, and in turn the reduction in the level of free, bioactive testosterone. These changes are often associated with sexual dysfunction in men (Rattya et al. 2001; Isojarvi et al. 2005). Additionally, an impairment of sexual health was also noted in men who were treated with clonazepam (CZP) (Cohen and Rosenbaum 1987; Fossey and Hamner 1994; Nardi et al. 2011). More safe therapies seem to rely on second-generation AEDs, like oxcarbazepine (OCX), gabapentin (GBP), levetiracetam (LEV) and lamotrigine (LTG). Although their influence on sexual functions in men has not been investigated extensively as yet, some improvement of sexual functioning during treatment with these drugs was noted (Husain et al. 2000; Schwartz et al. 2007; Luef et al. 2009).

Co-existence of epilepsy and erectile dysfunctions in male epileptic patients requires safe and effective medication. Sildenafil has been recognized as adequate in the treatment of erectile dysfunctions in men with epilepsy. However, according to recent reports, sildenafil might affect convulsant activity in both humans (Gilad et al. 2002) and in animal models of seizures (Riazi et al. 2006; Nieoczym et al. 2010a, 2010b; Montaser-Kouhsari et al. 2011). In addition, co-administration of sildenafil with AEDs might lead to the pharmacodynamic and/or pharmacokinetic interactions between these medications and to the reduction of the therapeutic activity of AEDs (Nieoczym et al. 2010a).

The above-mentioned facts prompted us to examine the effects of sildenafil on the threshold for clonic seizures in the subcutaneous s.c. PTZ test in mice and on the anticonvulsant efficacy of ethosuximide (ETS), CZP, VPA, PB and tiagabine (TGB) in this test. The s.c. PTZ-induced seizures are generally accepted as a model for myoclonic absence convulsions (Löscher and Schmidt 1988). The studied AEDs were selected on the basis of diverse mechanisms of their action. Furthermore, the acute adverse effects of sildenafil and its combination with the studied AEDs were investigated in the chimney test (impairment of motor performance), grip-strength test (changes in muscle strength) and step-through type of passive-avoidance task (disturbances in long-term memory) in mice. Total brain AEDs’ concentrations were measured to estimate pharmacokinetic interactions between sildenafil and the studied AEDs.

## Materials and methods

### Animals

Male albino Swiss mice weighing 24–30 g were used throughout this study after 1 week of acclimatization. The animals were housed in a colony in polycarbonate cages under strictly controlled housing conditions (ambient temperature 20–23°C, relative humidity 45–55%, 12-/12-h light/dark cycle, light on at 6:00 a.m., chow pellets and tap water continuously available). Mice were randomly divided into experimental groups. Each mouse was used only once. All experiments were conducted between 9:00 a.m. and 4:00 p.m. The total number of mice used in this study was 1,150.

The experimental protocol was approved by the First Local Ethics Committee at the Medical University of Lublin and all procedures were in strict compliance with the European Communities Council Directive of 24 November 1986 (86/609/EEC). Additionally, all efforts were made to minimize animal suffering and to use only the number of animal necessary to produce reliable scientific data.

### Drugs

The following drugs were used in this study: sildenafil [1-((3-(4,7-dihydro-1-methyl-7-oxo-3-propyl-1H-pyrazolo(4,3-d)pyrimidin-5-yl)-4-ethoxyphenyl)sulfonyl)-4-methylpiperazine citrate; kindly provided by Fako İlaçları AŞ, Istanbul, Turkey], ETS (Sigma, St. Louis, MO, USA), CZP (Clonazepamum, Polfa, Warsaw, Poland), VPA (as sodium salt; Sigma), PB (Polfa, Cracow, Poland), TGB (Gabitril, Sanofi Winthrop, Gentilly, France). Sildenafil, VPA, and PB were dissolved in saline, and the remaining drugs were suspended in 1% solution of Tween 80 (POCH, Gliwice, Poland) in saline. All drug solutions and suspensions were prepared freshly and administered intraperitoneally (i.p.) in a volume of 10 ml/kg body weight. TGB and CZP were administered 15 min, sildenafil and VPA 30 min, ETS 45 min, and PB 60 min prior to the tests. The pretreatment times were taken from the literature and were confirmed by our previous and pilot studies. The control animals received respective vehicles at the appropriate volume.

### Threshold for PTZ-induced clonic seizures

The threshold for clonic convulsions was determined in control (saline-treated) and sildenafil-treated (doses ranging from 5 to 40 mg/kg) mice by administration of PTZ (Sigma, Sigma-Aldrich Sp. z o.o., Poznań, Poland) at doses ranging from 50 to 100 mg/kg. PTZ was dissolved in saline and s.c. administered in a volume of 10 ml/kg into a loose fold of skin on the back of the neck of the animals (Löscher et al. 1991). Immediately after PTZ injection, animals were placed separately into transparent Plexiglas cages and observed for 30 min for the occurrence of clonic seizures. Clonic seizures were defined as clonus phase of generalized seizures of whole body and all four limbs lasting for at least 3 s, with loss of righting reflex. The number of animals with clonic seizures out of the total number of animals in each tested group was noted. Median convulsive dose (CD_50_) of PTZ, i.e., the dose that produced clonic seizures in 50% of the mice tested, was determined to evaluate the convulsive action of PTZ. To determine the CD_50_ value for control and sildenafil-treated animals, at least four varying doses of PTZ were tested and each experimental group consisted of eight mice. Afterward, the CD_50_ value was calculated from the equation of dose–response curve of PTZ, according to the method described by Litchfield and Wilcoxon (1949).

### PTZ-induced seizures in mice

The anticonvulsant activity of AEDs alone and in combination with sildenafil was investigated in the PTZ-induced clonic seizure test in mice (Löscher et al. 1991). Clonic convulsions were produced by s.c. injection of PTZ at a dose of 96.1 mg/kg, equal to its CD_97_ value (the dose that induced clonic seizures in 97% of animal tested). Following the PTZ administration, mice were placed separately into transparent Plexiglas cages and observed for clonic seizures (as described in the previous section) occurrence. Convulsant activity was recorded for 30 min after PTZ injection. The number of animals with clonic seizures out of the total number of mice tested was noted for each treatment condition. The anticonvulsant activity of each AED alone and in combination with sildenafil was evaluated as its ED_50_ (median effective dose, i.e., dose of the AED that protected 50% of the mice against PTZ-induced clonic convulsions). ED_50_ values were calculated according to the log-probit method described by Litchfield and Wilcoxon (1949) and their 95% confidence limits were transformed to standard errors of the mean (SEM). ED_50_ values were analyzed with one-way analysis of variance (ANOVA) followed by the Tukey post hoc test for multiple comparisons.

### Chimney test

The effects of sildenafil and its combinations with AEDs on motor performance were determined in the chimney test (Boissier et al. 1960). In this test, the inability of mice to climb backward up through a Plexiglass tube (3 cm, inner diameter × 30 cm, length) within 60 s is an indication of motor impairment. The results were presented as percentage of impaired mice in the group. Each group consisted of eight animals and the Fisher’s exact probability test was used for statistical comparisons.

### Grip-strength test

The effects of sildenafil and its combinations with the studied AEDs on skeletal muscular strength in mice were quantified by the grip-strength test (Meyer et al. 1979). The grip-strength apparatus (BioSeb, Chaville, France) comprised a steel wire grid (8 × 8 cm) connected to an isometric force transducer. The mice were lifted by their tails so that they could grasp the grid with their forepaws. The mice were then gently pulled backward by the tail until they released the grid. The measurements were carried out three times and the mean force exerted by each mouse before losing its grip was recorded. The mean force (± SEM) of eight animals per group was determined and expressed in grams-force (gf). Statistical analysis of the data was performed with one-way ANOVA followed by the Bonferroni’s post hoc test for multiple comparisons.

### Step-through passive-avoidance task

The step-through passive-avoidance task is widely accepted as an experimental paradigm for testing long-term memory (Venault et al. 1986). Animals were treated with sildenafil and with combinations with the studied AEDs. All drugs were administered before the training session. The apparatus used in the light–dark, step-through type of passive-avoidance protocol consisted of two boxes, one lit (10 × 13 × 15 cm) and the other dark (25 × 20 × 15 cm), connected via a guillotine door and equipped with an electric grid floor. During the training session, each mouse was gently placed in the light compartment of the apparatus and allowed to enter the dark room. After entering from the light room to the dark one, mice were punished by a 0.6-mA foot shock for 2 s. The mice that did not enter the dark compartment within 180 s were excluded from the experiment. The second session was carried out 24 h after the training session. The mice were placed in the light compartment and the retention time, i.e., the time that the mice took to enter the dark box, was noted. The median latencies with 25th and 75th percentiles were calculated in each group. Mice that did not enter the dark compartment for 180 s were considered to remember the task. Statistical analysis of data was performed with the nonparametric Kruskal–Wallis ANOVA.

### Measurement of total brain AED concentration

The measurement of total brain concentrations of the studied AEDs was undertaken at doses of the AEDs corresponding to their ED_50_ values in combination with sildenafil (40 mg/kg) from the s.c. PTZ-induced clonic seizure test in mice. Mice were killed by decapitation at times chosen to coincide with that scheduled for the PTZ test and the whole brains of mice were removed from skulls, weighed, harvested and homogenized in distilled water (1:2, w:v). The homogenates were centrifuged at 10,000 rpm for 10 min. Supernatants containing PB, ETS and VPA were analyzed by fluorescence polarization immunoassay using a TDx analyzer and dedicated reagents (Abbott Laboratories, North Chicago, IL, USA).

TGB and CZP concentrations were measured using an automated HPLC system (Dionex, Sunnyvale, CA, USA) consisting of a P580 LPG pump and UV/Vis detector (UVD 340S) and manual injector. Supernatants (500 μl) containing TGB were mixed with 100 μl of water and 100 μl of chloramphenicol (10 ng/ml in methanol/water (50:50) solution) and centrifuged at 3,000 rpm for 15 min. After centrifugation, 200 µl of the supernatant was pipetted into a C8 SPE column conditioned with 2 ml of water and solution of water/methanol in a ratio of 95:5 (v:v) and eluted with 1 ml of methanol. The eluant was evaporated to dryness and the residue was dissolved in 200 µl of methanol. This solution (20 µl) was injected into the HPLC system. The mobile phase comprised 20 mM phosphate buffer (pH 7):methanol:acetonitrile in a ratio of 480:260:260 (v:v:v). Chromatographic separation was achieved using Hypersil ODS-2 column. The brain homogenates (500 µl) containing CZP were mixed with 500 µl of 0.2 M carbonate buffer and 100 µl of chloramphenicol (10 ng/ml in methanol/water (50:50) solution), and subsequently 5 ml of hexane:ethyl acetate mixture in a ratio of 7:3 (v:v) was added. The samples were shaken out for 10 min and then centrifuged at 10,000 rpm for 15 min. Supernatant was transferred into a new tube and evaporated. The residue was dissolved in 200 µl of methanol and injected into the HPLC system. Chromatographic analysis was conducted using Zorbax SB-C18 column. The mobile phase comprised 40 mM triethylammonium phosphate buffer:acetonitrile (660:340, v:v).

The total brain AED concentration was expressed in μg/g (VPA, PB and ETS) or ng/g (CZP and TGB) of wet tissue as mean ± SEM of eight (VPA, PB and ETS) or ten (CZP and TGB) separate determinations. The data were compared by means of the unpaired Student’s *t* test.

## Results

### Effect of sildenafil on PTZ-induced clonic seizures and on the protective activity of AEDs against the clonic phase of PTZ-induced seizures in mice (Table 1)

In control (saline-treated) group, CD_50_ and CD_97_ doses of PTZ were calculated and were determined to be 71.7 and 96.1 mg/kg, respectively. Sildenafil administered i.p. at doses ranging from 5 to 40 mg/kg did not significantly affect threshold for PTZ-induced clonic seizures in mice [*F*(4, 83) = 0.533; *p* = 0.712].

**Table 1:** Influence of sildenafil on the threshold for PTZ-induced seizures in mice

Treatment (mg/kg)	CD_50_ of PTZ (mg/kg)	*n*	SEM
Control	71.70 (61.6–83.5)	8	5.57
Sildenafil (5)	69.12 (63.2–75.6)	24	3.15
Sildenafil (10)	67.61 (63.9–71.5)	24	1.93
Sildenafil (20)	71.96 (64.5–80.2)	16	4.63
Sildenafil (40)	64.92 (56.4–74.7)	16	4.64

Data are presented as median convulsive doses of PTZ (CD_50_: the dose which produces clonic seizures in 50% of animal tested) with 95% confidence limits in parentheses. Sildenafil was administered i.p. 30 min prior the s.c. administration of PTZ. CD_50_ values were calculated according to the log-probit method described by Litchfield and Wilcoxon (1949). One-way ANOVA was used to compare CD_50_ values; *F*(4, 83) = 0.533; *p* = 0.712. *n* total number of animals at those doses of PTZ whose convulsant effect was between 4 and 6 probits. SEM, standard error of the CD_50_ values

### Influence of sildenafil on the anticonvulsant activity of selected AEDs in the s.c. PTZ-induced seizure test in mice (Table 2)

Sildenafil (doses ranging from 10 to 40 mg/kg) administered in combination with CZP, VPA, PB and TGB did not change their anticonvulsant activity in the PTZ-induced seizure test in mice (*p* > 0.05). In contrast, sildenafil given concomitantly with ETS dose dependently enhanced its anticonvulsant effect against PTZ-induced clonic seizures [*F*(3, 99) = 17.540; *p* < 0.001]. Sildenafil at a dose of 20 mg/kg co-administered with ETS decreased its ED_50_ from 155.8 (139.7–173.7) mg/kg in the control group to 120.3 (101.0–143.2) mg/kg (22.8%; *p* = 0.02). The highest tested dose of sildenafil (40 mg/kg) was able to decrease ED_50_ of ETS to 72.29 (58.59–89.20) mg/kg, i.e., by 53.6% (*p* < 0.001).

**Table 2:** Influence of sildenafil on the anticonvulsant activity of selected AEDs in the s.c. PTZ seizure test in mice

Treatment (mg/kg)	ED_50_ (mg/kg)	*n*	SEM
CZP + saline	0.022 (0.015–0.032)	24	0.0042
CZP + sildenafil (10)	0.015 (0.011–0.020)	32	0.0023
CZP + sildenafil (20)	0.020 (0.016–0.024)	28	0.0020
CZP + sildenafil (40)	0.013 (0.010–0.019)	23	0.0021
VPA + saline	121.4 (104.9–140.5)	32	9.04
VPA + sildenafil (10)	127.4 (107.2–151.4)	24	11.21
VPA + sildenafil (20)	121.0 (102.3–143.3)	47	10.41
VPA + sildenafil (40)	101.0 (82.4–123.8)	24	10.50
PB + saline	12.68 (10.62–15.13)	28	1.15
PB + sildenafil (10)	11.83 (9.04–15.47)	27	1.62
PB + sildenafil (20)	13.94 (12.30–15.80)	32	0.89
PB + sildenafil (40)	13.03 (11.08–15.33)	24	1.08
TGB + saline	0.88 (0.61–1.25)	32	0.16
TGB + sildenafil (10)	0.86 (0.62–1.20)	16	0.14
TGB + sildenafil (20)	0.74 (0.58–0.97)	32	0.10
TGB + sildenafil (40)	0.75 (0.59–0.94)	24	0.09
ETS + saline	155.8 (139.7–173.7)	31	8.66
ETS + sildenafil (10)	140.4 (127.6–154.4)	24	6.83
ETS + sildenafil (20)	120.3 (101.0–143.2)^a^	24	10.73
ETS + sildenafil (40)	72.29 (58.59–89.20)^b^	24	7.76

Data are presented as median effective doses of AEDs (ED_50_: the dose which protects 50% of animal tested from PTZ-induced clonic seizures) with 95% confidence limits in parentheses. PTZ was administered s.c. at a dose of 96.1 mg/kg, which was its CD_97_ (a dose of PTZ provoking clonic convulsions in 97% of animal tested). ED_50_ values were calculated according to the log-probit method described by Litchfield and Wilcoxon (1949). One-way ANOVA was used to compare ED_50_ values; CZP *F*(3, 103) = 2.017; *p* = 0.116; VPA *F*(3, 123) = 0.904; *p* = 0.441; PB *F*(3, 107) = 0.722; *p* = 0.541; TGB *F*(3, 100) = 0.309; *p* = 0.819; ETS *F*(3, 99) = 17.540; *p* < 0.001. *n* total number of animals at those doses of AEDs whose anticonvulsant effect was between 4 and 6 probits; SEM, standard error of the mean of ED_50_ values; ^a^ *p* < 0.05; ^b^ *p* < 0.001 versus AED + saline-treated group

### Brain AEDs’ concentration (Table 3)

Sildenafil administered at a dose of 40 mg/kg did not affect significantly total brain concentration of the AEDs tested. Despite the fact that sildenafil increased anticonvulsant activity of ETS in the s.c. PTZ test in mice, it did not influence the concentration in the brain.

**Table 3:** Influence of sildenafil on total brain concentration of AEDs in mice

Treatment (mg/kg)	Brain concentration
CZP (0.014) + saline	14.99 ± 0.38 ng/g
CZP (0.014) + sildenafil (40)	14.13 ± 0.85 ng/g
VPA (101.0) + saline	619.7 ± 18.61 µg/g
VPA (101.0) + sildenafil (40)	611.8 ± 28.68 µg/g
PB (13.03) + saline	45.74 ± 0.72 µg/g
PB (13.03) + sildenafil (40)	45.87 ± 0.79 µg/g
TGB (0.75) + saline	115.6 ± 5.8 ng/g
TGB (0.75) + sildenafil (40)	123.0 ± 6.3 ng/g
ETS (72.3) + saline	134.9 ± 7.73 µg/g
ETS (72.3) + sildenafil (40)	153.5 ± 7.99 µg/g

Data are presented as the mean ± standard error of the mean (SEM) of at least eight separate brain preparations. Sildenafil and the studied AEDs were administered i.p. at times scheduled from the s.c. PTZ test and in the highest tested doses of sildenafil. The results were statistically compared with Student’s *t* test; CZP *t* = 0.9269; *df* = 18, *p* > 0.05; VPA *t* = 0.229; *df* = 14; *p* > 0.05; PB *t* = 0.1231; *df* = 14; *p* > 0.05; TGB *t* = 0.8641; *df* = 18; *p* > 0.05; ETS *t* = 1.672; *df* = 14; *p* > 0.05

### Effects of sildenafil alone and in combination with selected AEDs on muscular strength, motor performance and long-term memory in mice (Table 4)

One-way ANOVA revealed that neither sildenafil at doses of 20 and 40 mg/kg nor its combination with all the studied AEDs significantly altered muscular strength in mice, as assessed by the grip-strength test [*F*(12, 91) = 0.8465; *p* = 0.603]. Likewise, sildenafil administered alone and combined with AEDs did not change significantly motor coordination (*p* > 0.05 versus control group), as determined in the chimney test, and long-term memory in mice (*KW* = 12.08; *p* = 0.4395), as assessed by the step-through passive-avoidance task.

**Table 4:** Effects of sildenafil and its combination with selected AEDs on motor performance, muscular strength and long-term memory in mice

Treatment (mg/kg)	Motor impairment (%)	Retention time (s)	Muscle strength (gf)
Saline	0	180 (180; 180)	107.7 ± 5.5
Sildenafil (20)	0	180 (180; 180)	108.5 ± 5.8
Sildenafil (40)	0	180 (180; 180)	118.1 ± 6.9
CZP (0.02) + sildenafil (20)	0	180 (180; 180)	101.9 ± 5.1
CZP (0.02) + sildenafil (40)	0	180 (180; 180)	102.0 ± 6.9
VPA (121.05) + sildenafil (20)	0	180 (112.5; 180)	101.0 ± 2.5
VPA (101.0) + sildenafil (40)	0	180 (85.5; 180)	106.9 ± 5.7
PB (13.94) + sildenafil (20)	0	180 (99.75; 180)	109.2 ± 4.3
PB (13.03) +sildenafil (40)	0	180 (180; 180)	103.5 ± 5.1
TGB (0.75) + sildenafil (20)	0	180 (180; 180)	102.3 ± 4.3
TGB (0.75) + sildenafil (40)	0	180 (180; 180)	104.5 ± 4.9
ETS (120.26) + sildenafil (20)	0	180 (171; 180)	111.8 ± 4.8
ETS (72.29) + sildenafil (40)	0	180 (82.5; 180)	109.0 ± 5.0

Results are presented as percentage of animals showing motor coordination impairment in the chimney test in mice, as median retention times (in s; with 25th and 75th percentiles in parentheses) from the step-thorough type of passive-avoidance task, assessing long-term memory in mice, and as mean (± SEM) grip-strength in grams-force (gf) from the grip-strength test, assessing neuromuscular strength in mice. The Fisher’s exact probability test was used to analyze the results from the chimney test. Statistical analysis of data from the grip-strength test was performed with one-way ANOVA: *F*(12, 91) = 0.8465; *p* = 0.603. The results obtained in the passive-avoidance test were analyzed with nonparametric Kruskal–Wallis ANOVA: *KW* = 12.08; *p* = 0.4395. Each experimental group consisted of eight animals. All drugs were administered i.p. at times scheduled from the PTZ test and at doses corresponding to their ED_50_ values against clonic seizures

## Discussion

The effects of sildenafil, a selective PDE5 inhibitor, in experimental models of epileptic seizures were recently extensively investigated. It was reported that sildenafil had anticonvulsant effect against seizures induced by electric stimulation, like electroshock in mice (Nieoczym et al. 2010a) and amygdala-kindling in rats (Nieoczym et al. 2010b), while its proconvulsant activity was noted in models of seizures induced by γ-aminobutyric acid (GABA) antagonists, like PTZ (Riazi et al. 2006; Gholipour et al. 2009; Nieoczym et al. 2010b; Montaser-Kouhsari et al. 2011) and bicuculline (Riazi et al. 2006). Sildenafil did not influence cocaine-induced seizures, which are mediated by other mechanisms than GABAergic system (Nieoczym et al. 2009). With respect to PTZ-induced seizures, it is interesting to note that sildenafil decreases seizure threshold when PTZ is administered intravenously (i.v.; timed infusion) (Riazi et al. 2006; Gholipour et al. 2009; Nieoczym et al. 2010b) and does not affect seizure threshold when PTZ is given s.c. (present study). This may point to marked differences between these two modes of seizure induction by PTZ. Furthermore, it was reported that sildenafil abolished anticonvulsant effects of adenosine (Akula et al. 2008), diazepam (Gholipour et al. 2009) and lithium chloride (Bahremand et al. 2010) in the timed i.v. PTZ infusion test in mice.

It seems that the influence of sildenafil on seizure activity in the i.v. PTZ test is strictly connected to the NO/cGMP pathway. NO is synthesized enzymatically by nitric oxide synthase (NOS) from amino acid l-arginine and activates GC by binding with its heme group. Its activation leads to the increase in the concentration of the second messenger, cGMP. It is known that cGMP might affect the activity of protein kinases and cGMP-gated cation channels. The additional cellular targets for cGMP are PDEs, i.e., PDE5, which transform this cyclic nucleotide to its linear form, guanosine-5′-monophosphate (GMP). The level of cGMP is dependent on both GC and PDEs activity. NO might not only act as a signaling molecule in the central nervous system by itself, but might also influence excitatory and inhibitory neurotransmission by NO/cGMP pathway (Esplugues 2002). Furthermore, interactions between NO/cGMP pathway and neurotransmitter systems are bidirectional because synthesis of NO is enhanced by *N*-methyl-d-aspartate (NMDA)-induced Ca^2+^-influx (Prast and Philippu 2001) and, on the other hand, cGMP activates protein kinases which phosphorylate subunits in the GABA_A_ receptor complex, thereby reducing their activity (Ahern et al. 2002). Furthermore, it was reported that a high level of NO in the central nervous system limited glutamate reuptake into neurons (Bogdanov and Wurtman 1997). On the other hand, increase in the cGMP level might enhance release of glutamate (Prast and Philippu 2001). Maintaining of balance between excitatory and inhibitory processes in the central nervous system is essential in normal brain functioning and preventing epileptic disorders. Participation of the NO/cGMP pathway in the neurotransmission processes attests to possibility of influence of sildenafil on seizure activity.

Results of the previous studies point to the fact that influence of sildenafil on seizure activity in experimental models of epileptic seizures is mediated by NO/cGMP pathway, because NOS inhibitor, *N* ^G^-nitro-l-arginine methyl ester (l-NAME), and GC inhibitor, methylene blue (MB), inhibit proconvulsant activity of the studied PDE5 inhibitor, while NOS substrate, l-arginine, and NO donor, sodium nitroprusside show additive proconvulsant effect (Riazi et al., 2006). Furthermore, results presented by Montaser-Kouhsari et al. (2011) revealed that the proconvulsant effect of sildenafil in the PTZ seizure test was also mediated by the opioid system, because combination of sub-effective doses of sildenafil and morphine significantly decreased seizure threshold for PTZ-induced clonic seizures in mice, while an opioid µ-receptor antagonist, naltrexone, prevented proconvulsant activity of sildenafil.

In our present study, sildenafil did not influence threshold for clonic seizures induced by s.c. administration of PTZ in mice. PTZ-induced seizures, both after i.v. and s.c. administration, are generally accepted methods for evaluating the therapeutic effect of substances on seizure activity and they are mediated by blocking of GABAergic neurotransmission in the central nervous system, but it is well known that the s.c. PTZ seizure test is less sensitive than the timed i.v. PTZ infusion paradigm. It might be supposed that the lack of effect of sildenafil in our research eventuated from the little sensitivity of the used seizure test.

Despite the lack of effect on threshold for clonic seizures induced by s.c. administration of PTZ in mice, sildenafil enhanced anticonvulsant activity of ETS but did not influence CZP, VPA, PB and TGB protective action in this test. The effect of sildenafil on the activity of selected AEDs was investigated previously in the maximal electroshock (MES) test in mice. It was noted that sildenafil enhanced anticonvulsant activity of CBZ, VPA and TPM against MES-induced seizures, although interactions with CBZ and VPA were pharmacokinetic in nature (Nieoczym et al. 2010a). An increase in protective activity of ETS in the present study seems to be related to the pharmacodynamic rather than pharmacokinetic interactions, because sildenafil did not change the total brain concentration of ETS in mice. The principal mechanism of anticonvulsant action of ETS is strictly connected to the blockade of T-type calcium channels (Rogawski and Löscher 2004; Lasoń et al. 2011). It seems that pharmacodynamic interactions between sildenafil and ETS in the present study were not related to the direct influence of high level of cGMP on T-type calcium channels, because it was reported that an analog of cGMP, 8-*p*-chlorophenylthio-cGMP, significantly increased the peak amplitude of a T-type calcium current in the newt olfactory receptor cells (Kawai and Miyachi 2001). It is possible that these interactions were connected to the indirect influence of sildenafil on L-type calcium channels. Kim et al. (2010) noted antinociceptive effect of sildenafil in the formalin test in mice and postulated that this effect was mediated by increased cGMP level and activation of PKG, which is the major intracellular receptor protein for cGMP. Activation of PKG is followed by L-type calcium channel inhibition. Blockade of L-type calcium channels is accepted as one of the mechanisms of action of AEDs and it was noted in case of CBZ, felbamate and TPM (Lasoń et al. 2011). Furthermore, the effect of sildenafil on ETS activity might also arise out of its interactions with the GABAergic system. Experiments carried out by Huang et al. (2010) revealed that antinociceptive effect of sildenafil in the neuropathic pain model in rats was mediated by enhancement of GABAergic neurotransmission. Seen as a whole, sildenafil-induced increase in anticonvulsant activity of ETS seems to be a result of their interactions with different cellular targets, i.e., T- and L-type calcium channels and GABAergic system.

Neither sildenafil alone nor its combinations with the studied AEDs cause any acute side effects as demonstrated in the chimney test and the step-through passive-avoidance task in mice. Furthermore, previous studies reported that AEDs administered at doses which protected 50% of mice from the clonic convulsions in the s.c. PTZ test did not impair motor coordination and long-term memory in mice in these experimental paradigms (Łuszczki et al. 2005; Łuszczki et al. 2006; Lasoń et al. 2011). The grip-strength test did not reveal any changes in the muscular strength caused by a combination of sildenafil with the studied AEDs.

In conclusion, our data showed that sildenafil did not significantly affect threshold for clonic seizures induced by s.c. administration of PTZ in mice. These results replenish previous reports about both pro- and anticonvulsant action of sildenafil in animal models of epileptic seizures. Additionally, sildenafil enhanced anticonvulsant activity of ETS in the s.c. PTZ test in mice without any significant changes in the brain concentration of the AED, which suggested that the interaction between these drugs was pharmacodynamic in nature. In contrast, sildenafil did not generate any changes in the anticonvulsant activity of CZP, VPA, PB and TGB. The results of the present study suggest that combination of ETS with sildenafil in epileptic male patients with co-existing erectile dysfunctions might be safe and beneficial. The mechanism of pharmacodynamic interactions between sildenafil and ETS should be carefully evaluated in other experimental models of seizures and epilepsy.
